# CCR5 signalling, but not DARC or D6 regulatory, chemokine receptors are targeted by herpesvirus U83A chemokine which delays receptor internalisation via diversion to a caveolin-linked pathway

**DOI:** 10.1186/1476-9255-6-22

**Published:** 2009-07-30

**Authors:** Julie Catusse, David J Clark, Ursula A Gompels

**Affiliations:** 1 Pathogen Molecular Biology Unit, Department of Infectious & Tropical Diseases, London School of Hygiene & Tropical Medicine, University of London, Keppel St, London WC1E 7HT, UK

## Abstract

**Background:**

Herpesviruses have evolved chemokines and chemokine receptors, which modulate the recruitment of human leukocytes during the inflammatory response to infection. Early post-infection, human herpesvirus 6A (HHV-6A) infected cells express the chemokine receptor U51A and chemokine U83A which have complementary effects in subverting the CC-chemokine family thereby controlling anti-viral leukocyte recruitment. Here we show that, to potentiate this activity, the viral chemokine can also avoid clearance by scavenger chemokine receptors, DARC and D6, which normally regulate an inflammatory response. Conversely, U83A delays internalisation of its signalling target receptor CCR5 with diversion to caveolin rich membrane domains. This mechanism can redirect displaced human chemokines to DARC and D6 for clearance of the anti-viral inflammatory response, leaving the viral chemokine unchecked.

**Methods:**

Cell models for competitive binding assays were established using radiolabeled human chemokines and cold U83A on CCR5, DARC or D6 expressing cells. Flow cytometry was used to assess specific chemotaxis of CCR5 bearing cells to U83A, and internalisation of CCR5 specific chemokine CCL4 after stimulation with U83A. Internalisation analyses were supported by confocal microscopy of internalisation and co-localisation of CCR5 with caveosome marker caveolin-1, after virus or human chemokine stimulation.

**Results:**

U83A displaced efficiently human chemokines from CCR5, with a high affinity of 0.01nM, but not from DARC or D6. Signalling via CCR5 resulted in specific chemoattraction of primary human leukocytes bearing CCR5. However, U83A effective binding and signalling to CCR5 resulted in delayed internalisation and recycling up to 2 hours in the absence of continual re-stimulation. This resulted in diversion to a delayed caveolin-linked pathway rather than the rapid clathrin mediated endocytosis previously shown with human chemokines CCL3 or CCL4.

**Conclusion:**

U83A diverts human chemokines from signalling, but not regulatory or scavenger, receptors facilitating their clearance, while occupying signalling receptors at the cell surface. This can enhance virus specific inflammation, facilitating dissemination to replication sensitive leukocytes while evading clearance; this has implications for linked neuro-inflammatory pathologies.

## Background

Human herpesvirus 6 (HHV-6) is a wide-spread blood-borne virus, causing common childhood infections, resulting in febrile disease with occasional rash (*Exanthem Subitum*) and further serious complications, including encephalitis [1]. There are two variants, HHV-6A and B; HHV-6A has been linked with further neuro-inflammatory disease including multiple sclerosis (MS) and encephalopathy. HHV-6 is predominantly lymphotropic and has evolved mechanisms for the dysregulation of human immunity including diversion of chemokine activities. Chemokines interact with defined receptors expressed on specific leukocyte subsets, resulting in their activation and migration (chemotaxis) toward a chemokine gradient created by secretion from infected or damaged cells. Hence, chemokines are involved in hematopoietic cell traffic, inflammation and virus immunity as they can attract antigen presenting cells to sites of infection, mediate lymph node homing or activate immune defence mechanisms. To overcome the chemokine defence mechanism and redirect it towards enhanced virus persistence, HHV-6 encodes two chemokine receptors (U12 and U51) and one chemokine (U83) [2-5]. These are potential virulence factors in linked inflammatory pathologies. Furthermore, U83 is the only HHV6-specific hypervariable gene, and therefore key for biological differences between HHV-6 A and B strains. Laboratory adapted strains can have mutations affecting U83 expression, but both wild-type variants can encode signal sequences mediating chemokine secretion [2]. U83A from HHV-6A is a high affinity broad-range yet selective agonist for CC-chemokine receptors CCR1, CCR4, CCR5, CCR6 and CCR8, while HHV-6B U83B is a low affinity CCR2 ligand [2,4]. This disparity suggests U83 plays a key role in tropism and pathology differences between variant strains. Moreover, recent reports demonstrate HHV-6 integrations in the germ line of approximately 1% of the population [6,7], thereby giving expression of U83 the potential to exhibit as a human chemokine allele, not only from widespread latent infection, but also as part of the human genomic complement. Thus, it is important to establish effects of U83A in an inflammatory response.

At early times post-infection, both viral chemokine U83A and chemokine receptor U51A are expressed and exert thorough regulation of the human CC-chemokine system by time-controlled specific agonism, antagonism and competition, (see Table 1) [2,8-10]. There are two versions of U83A, an immediate-early expressed spliced form, which leads to an N-terminal truncation, U83A-N, and a full length form, U83A, made later after virus replication, when splicing is suppressed. Both can bind chemokine receptors efficiently, but only U83A can signal [2,4,11]. Here, we demonstrate that U83A has developed the capacity to avoid clearance by scavenger chemokine receptors and to control signalling receptors activity by blocking their internalisation and addressing them to caveolin enriched membrane domains. Scavenger receptors are usually involved in regulation of effective chemokine levels and are required to dampen potentially damaging inflammatory responses driven by chemokines once an infection is cleared [12-14]. U83A modification of the human chemokine response is shown in this article to be broad and complex, as it can interfere with signalling receptor function as well as avoid scavenger receptor clearance, a mechanism that potentiates its own activity on signalling receptors.

**Table 1:** Human chemokines and their receptors targeted by early HHV-6A infection

HHV-6A protein	Bound* chemokines	Displaced* chemokines	Affected* signalling Receptors	Affected scavenger receptors
U51A receptor	CCL2		CCR2	DARC, D6
	CCL5		CCR1, 3, 5	DARC, D6**
	CCL7		CCR1, 2, 3	DARC, D6
	CCL13		CCR1, 2, 3	DARC, D6
	CCL11		CCR3	DARC, D6
	CCL19		CCR7	CCX-CKR
	XCL1		XCR1	-
				
**U83A chemokine		CCL3, 5	CCR1	DARC**, D6**
		CCL17, 22	CCR4	D6
		CCL3, 4, 5	CCR5	DARC**, D6**
		CCL20	CCR6	-
		CCL1	CCR8	-

*references [2,5,8-10], **: shown in this paper, -: unknown

There are three 'atypical' or scavenger chemokine receptors with roles in regulation of the chemokine system namely D6, DARC (Duffy antigen/receptor for chemokines) and CCX-CKR (chemocentryx chemokine receptor). Both D6 and DARC clear chemokines that bind signalling receptors CCR1, CCR2, CCR3, CCR4 and CCR5. In addition, DARC targets chemokines for receptors CXCR1 and CXCR2. CCX-CKR seems to have a more narrow spectrum, comprising chemokines which bind receptors CCR7, CCR9 and CXCR5 [12,15] (Table 1). Signalling receptors respond to human chemokines as well as viral chemokine U83A binding by inducing G-protein activation, increasing intracellular calcium levels, and various signalling cascades leading to cell polarisation, cytoskeletal changes, and chemotaxis. We have shown previously that U83A is able to block signalling receptor function by stopping their endocytosis via clathrin coated pits [8]. This effect was specific, since rapid clathrin-linked endocytosis of transferrin continued in the presence of U83A, and also CCL4 induced CCR5 in the absence of U83A. Here we show that U83 interferes with chemokine receptors activities via induction of their co-localisation rather with caveolin-1, in a delayed endocytic pathway. In contrast to signalling receptors, scavenger receptors bind chemokine efficiently, but do not induce known intracellular signalling pathways. Instead there is chemokine sequestration, internalisation, degradation, or re-localisation through transcytosis [12,15-17]. D6 and DARC bind chemokines with specificities similar to U83A, thus they are investigated here and compared to modulatory effects on CCR5 signalling.

## Methods

### Receptor binding

COS-7 cells transfected with indicated receptors as described [2] (1 × 10^6^), were incubated in Binding buffer (RPMI 1640, 0.1% BSA, and 20 mM HEPES, pH 7.4) for 2 h at 4°C with 125 pM of ^125^I radiolabelled chemokine (Perkin Elmer, specific activity: 2200 Ci/mM) in absence or presence of increasing concentrations of cold competitor viral (U83A or U83A-N) [2] or human chemokines (R&D Systems Europe Ltd., Abingdon, UK). After 2 h incubation on ice, cells were separated from unbound chemokine by microcentrifugation though a phthalate oil cushion (1.5 parts dibutylphthalate to 1 part bis(2-ethylhexyl)phthalate) as described [2,4], with bound radioactivity counted with a gamma counter. Data and statistical analyses used Prism 0.1.53 software (GraphPad).

### Internalisation assay

CCR5 expressing cells, MAGI-CCR5E [8], were incubated for 10 minutes at 37°C in absence or presence of 100 nM of U83A, washed in Binding buffer and incubated at 37°C and 5% CO_2 _for 30, 60 and 120 minutes before stimulation by 100 nM of CCL4 for 10 minutes or buffer only for unstimulated negative control. Cells were washed then resuspended in FACS buffer PBS, 0.1% BSA) and Fc blocked using 1 μg of human IgG/10^5 ^cells for 15 minutes at room temperature. Cells were then incubated at 4°C with fluorescein isothiocyanate, FITC, linked-CCR5 antibody (FAB 182F; R&D systems) for 30 minutes, washed three times with ice cold FACS buffer, fixed with 4% PFA and CCR5 surface expression determined as described [8] using a FACS calibur flow cytometer (BD Biosciences, Oxford, UK) and results analysed with FlowJo (Tree Star Inc.). Matching isotypes (mouse IgG2B) were used as negative controls and results are expressed as percentage of expression at time 0 for each treatment and subsequent incubation time.

### Chemotaxis assay

Chemotaxis was assayed using 96 well microchemotaxis chambers (ChemoTx, Neuroprobe, Gaithersberg, MD, USA) as described [8] with human donor peripheral blood mononuclear cells (PBMC) supplied from healthy laboratory volunteers, with local ethical committee approval, using anonymous coded samples (one donor per experiment). PBMC were purified as described [8] using EDTA anti-coagulated blood centrifuged over a Histopaque 1077 cushion (Sigma Aldrich, Irvine, UK) with cells collected from the interphase, then washed twice with phosphate buffered saline. Cells were resuspended in 10 ml RPMI, 10% fetal calf serum and used either immediately or after culture in ultra-low attachment tissue culture plasticware (Corning, NY, USA) for 3 days as described [8]. Chemokines were diluted in migration buffer, HBSS (Invitrogen, Paisley, UK) with 0.1% BSA (Sigma Aldrich, Irvine, UK) and added to the bottom chambers, including wells with buffer only negative control. A 5 μm filter was placed on top and cells resuspended in the same buffer were layered on the top filter membrane, then cultured for 1.5 hours at 37°C, 5% CO_2_. Cells were then gently wiped from the top membrane and the plate centrifuged for 2 minutes. Migrated cells in the bottom chambers were pooled from 8 wells per treatment and assayed for CCR2 and CCR5 expression by flow cytometry as above, with FITC-CCR5 antibody and phycoerythrin, PE, linked CCR2 antibody (FAB 151P) with isotype controls (FITC-mouse IgG2B IC004F and PE-mouse IgG2B IC004P) (R&D Systems). Chemotaxis assays were in triplicate from three independent assays of different donor cells.

### Confocal microscopy

As described previously [8], U373-MAGI-CCR5E cells were grown on coverslips for 24 hours then starved in serum-free medium for 30 minutes at 37°C, 5%CO_2_. After one washing in pre-warmed serum-free medium at 37°C, the cells were incubated with chemokines for 10 or 30 minutes. Cells were then permeabilized, by treatment with 0.05% saponin in 0.5% BSA-PBS for 10 minutes and then labelled with a buffer containing tetramethylrhodamine B thioisocyanate, TRITC-anti-human caveolin polyclonal antibody (Sc894, Santa Cruz Biotechnology Inc., CA, USA) and FITC-anti-human CCR5 monoclonal antibody (R&D Systems, UK) as described [8]. Next, the labelled cells were washed three times in ice-cold PBS containing 0.5% BSA, followed by fixation in 3% paraformaldehyde for 10 minutes. After three washings in ice-cold PBS containing 0.5% BSA, free aldehyde groups were quenched with 50 mM NH_4_Cl in PBS for 10 minutes. The coverslips were washed three times in PBS and then mounted using Vectashield mounting solution containing DAPI for nucleus detection (Vector Laboratories, Burlingame, CA, USA). Cells were examined using Z-stack sections (at 0.39-μm interval), and pictures acquired on a Zeiss LSM 510 Axioplan microscope with a Plan-Apochromat 63×/1.4 oil objective by an AxioCam with magnification ×630 under oil immersion (Zeiss, Jena, Germany). Digital images were analyzed with Zeiss LSM Image Browser, version 3.5.0.376 [EC] (AxioCam). Fluorochromes were excited at 488 nm for FITC and 542 nm for TRITC.

## Results

### U83A efficiently displaces human chemokines from CCR5 signalling chemokine receptor in model system to compare to regulatory receptors

**Figure 1:**
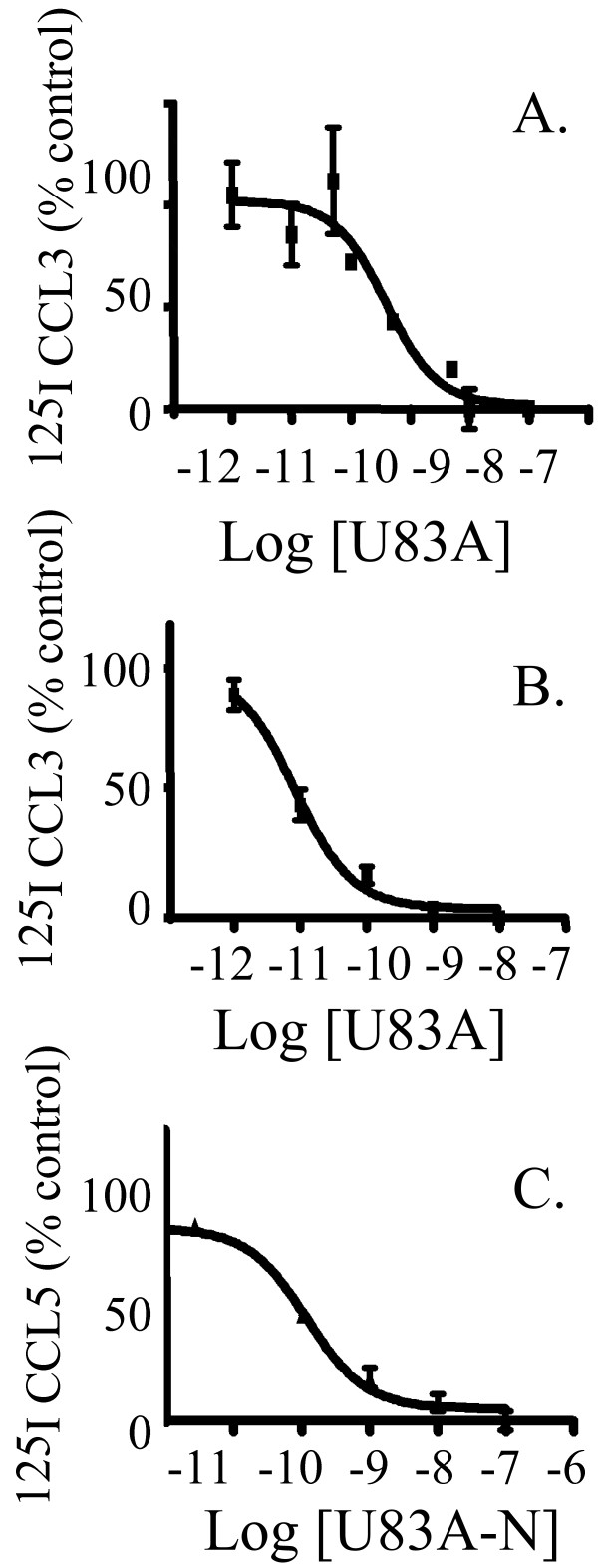
**Competitive binding of U83A to signalling receptors CCR1 and CCR5 displaces human chemokines**. COS-7 cells transfected with CCR1 (A) or CCR5 (B, C) were incubated with [^125^I]CCL3 (A and B) or [^125^I]CCL5 (C) in the presence of increasing concentration of cold chemokine. IC_50 _obtained were for (A) U83A: 0.4 nM, (B) U83A: 0.01 nM and (C) U83A-N: 0.1 nM. Binding curves were fitted by nonlinear regression and IC_50 _values were calculated using Graphpad Prism.

In order to investigate U83A activities on regulatory receptors and to compare them to those on signalling receptors, U83A binding competition was screened against chemokines also relevant for regulatory receptor specificity. U83A can displace human chemokines from receptors CCR1, CCR4, CCR5, CCR6 and CCR8. Of these, chemokines that bind CCR1, CCR4 and CCR5, can also be cleared by D6 and DARC (Table 1). Therefore, CCL3 and CCL5 were selected for the comparisons as their binding can be competed by U83A on signalling receptors CCR1 and CCR5 as shown in previous tests on CCR5 bearing astrocytic U373, monocytic U937, PBMC as well as model CCR1 or CCR5 transfected COS-7 cells [2,8]. Both CCL3 and CCL5 can also be cleared by D6 [18], and CCL5 modulated by DARC [14]. Competitive chemokine binding was tested using a model COS-7 cell system which we used previously to characterize U83A binding specificity [2,8], as high levels of receptor expression can be obtained in these monkey fibroblast cells without expression of endogenous human chemokine receptors. Cells were transiently transfected with a pcDNA3 vector containing the chemokine receptor gene of interest, and evaluated using control signalling receptors (CCR1, Figure 1A, and CCR5 Figure 1B, C). U83A displacement of radiolabelled CCL3 on signalling receptor CCR1, at 0.4 nM affinity, repeated previous findings and validated the model (Figure 1A). Highest affinity was observed for the full length U83A chemokine to CCR5, at 0.01 nM (Figure 1B). This was consistent with previous observations using CCL3 displacement on CCR5 expressing MAGI-CCR5 cells as well as COS-7 cells, of 0.011 nM and 0.03 nM, respectively. This confirmed the expression system for comparison to D6 and DARC activities. The CCR5 affinity also is the highest observed so far for the receptors interacting with U83A which also include CCR4, CCR6 and CCR8 (Table 1). In contrast, the spliced, truncated version of U83A, U83A-N, showed a 0.1 nM affinity for CCR5, in competition binding with CCL5 (Figure 1C). Thus, U83A-N displaces human chemokines less effectively than the full-length form. This is the first demonstration of U83A-N binding CCR5 using this cell expression system and also via CCL5 competition. It is consistent with the affinity of 8.3 nM observed in competition against CCL3 binding in U373-MAGI-CCR5 cells which also showed higher affinity binding with the full length molecule. Competition against CCL3 was lower in primary human leukocytes at 90 nM as well as U937 monocytic cell lines at 54 nM, but these express both the lower affinity receptor CCR1, as well as CCR5. However, U83A-N was more effective at competing CCL5 than previous results with CCL3, showing at least one log higher affinity (Figure 1C) [2,8]. These results tested efficiency of both human chemokine CCL3 and CCL5 displacements by U83A and U83A-N in this expression system, which then allowed comparisons to their binding on scavenger chemokines receptors and possible displacement by the viral chemokines.

### U83A does not displace human chemokines from DARC and D6 regulatory receptors

**Figure 2:**
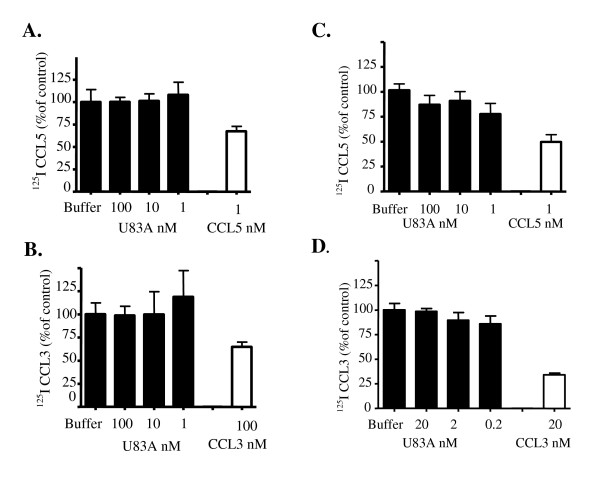
**U83A does not displace human chemokines binding to scavenger receptors D6 and DARC**. Cells transiently expressing D6 (A and B) or DARC (C and D) were used to investigate the binding of U83A to these receptors, monitored by displacement of [^125^I] CCL5 (A and C) or CCL3 (B and D). Positive controls were performed using cold forms of the radiolabelled chemokine, showing significant displacement of the radiolabelled form, (A) P < 0.05, (B) P < 0.01, (C and D) P < 0.001, unpaired T test.

U83A binding to regulatory chemokines receptors D6 and DARC was then investigated using this model. Relevant concentrations of the "cold" form of human chemokines were used as positive controls (Figure 2, white columns). CCL5 was used at the lowest dose inducing consistent displacement (1 nM) as higher doses have been shown to aggregate and interact with glycosaminoglycans inducing unusual binding profiles [19,20]. Competitive binding of this control confirms transfected receptor expression and specificity. 1 nM of CCL5 and 100 nM of CCL3 displaced respectively 33% and 35% of binding to D6 (Figure 2A and 2B) and 1 nM of CCL5 and 20 nM of CCL3 displaced respectively 51% and 66% of binding to DARC (Figure 2C and 2D). Interestingly, in DARC expressing K562 cells, CCL3 was described elsewhere as a weak DARC ligand relative to CCL5 [21,22]. Since only heterologous radiolabelled CXCL1 was used as a competitor in that report, CCL3 may bind to a different site on DARC only revealed by homologous displacement as shown here. We obtained consistent binding and homologous displacement of CCL3 and CCL5 to DARC, as well as D6 (Figure 2). However, U83A did not compete the binding of radiolabelled chemokines in any of the tested combinations or concentrations. D6 was monitored in competition against CCL5 or CCL3 (Figure 2A and 2B) and DARC binding by competition against CCL5 or CCL3 (Figure 2C and 2D). No competition was observed for U83A-N (not shown).

### U83A induces specific chemotaxis of human leukocytes bearing CCR5

**Figure 3:**
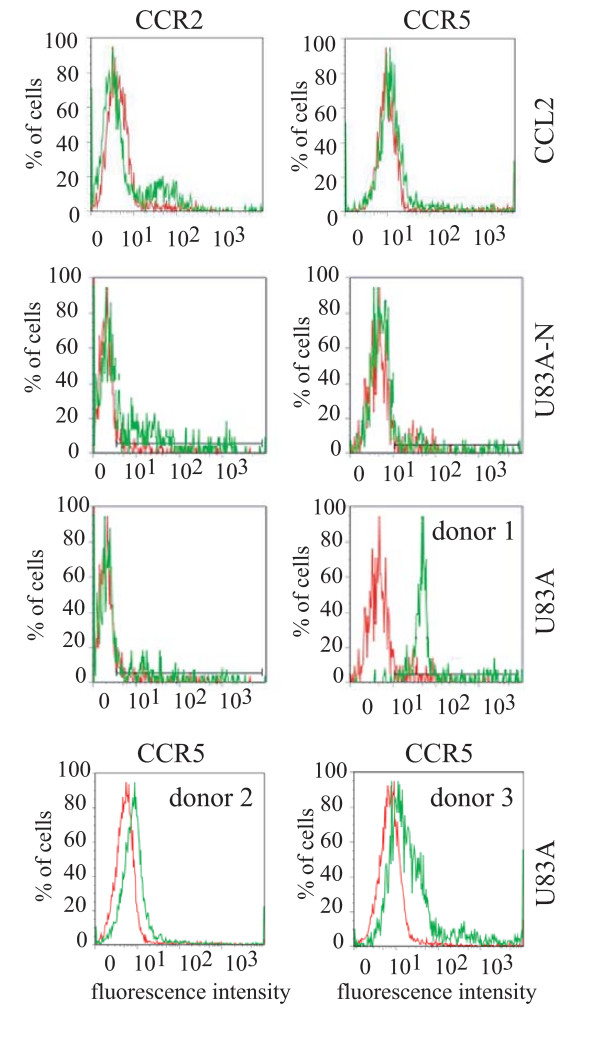
**U83A specifically chemoattracts CCR5 bearing primary human leukocytes**. PBMCs were plated out on a Neuroprobe chemotaxis apparatus. Lower chambers were filled with a range of chemokines. After 90 minutes incubation, cells remaining on top of the filter were removed and the migrated cells, in the wells below the filter, collected and stained for CCR2 and CCR5. Red peak denotes the background cell migration (buffer only) and green denotes the test peptide/chemokine. CCR2 antibody used was linked to PE and CCR5 antibody linked to FITC. The top, second and third panels show flow cytometry results for cells exposed to 1 nM of CCL2, U83A-N or U83A, respectively. Migration of CCR2 bearing cells were shown towards wells containing CCL2, and CCR5 bearing cells towards those with U83A. Experiments were repeated three times, one representative result shown. For the U83A treatment, the CCR5 specific results from two further donors are shown in the bottom panel. There was no specific chemotaxis detected using the negative control with migration buffer only.

In contrast to activities on the regulatory receptors, previous data show U83A binds CCR5 resulting in a signalling cascade which can lead to chemotaxis. Although chemotaxis using primary human peripheral blood mononuclear cells (PBMC) was demonstrated [8], the specificity of the migrated cells had not been confirmed. To test this, and to compare to the inability to interact with the scavenger receptors, U83A was used to stimulate primary human PBMC, from different donors, either cultured for three days to induce CCR5 or uncultured cells which primarily expressed CCR2. 1 nM concentrations were used based on optimum U83A chemotaxis indices as previously assayed using the microchemotaxis chambers, but without further analyses using flow cytometry [8]. In contrast, U83A-N did not mediate chemotaxis at concentrations up to 100 nM. Antibody staining and flow cytometry were then performed with the cells that had migrated towards the human or viral chemokines, in the bottom wells of the microchemotaxis chambers. Specific chemotaxis of cells bearing CCR5 but not CCR2, towards 1 nM U83A, was demonstrated, consistent with the binding specificity of U83A (Figure 3). In contrast, the human chemokine CCL2 showed specific chemotaxis for CCR2, but not CCR5. No specific chemotaxis was shown for U83A-N, consistent with lack of activity in chemotaxis assays without flow cytometry. Results for three separate donors treated with U83A are shown. These displayed varying responses, but all specific for CCR5. Thus, full length U83A stimulation can result in specific chemotaxis of CCR5 bearing cells.

### U83A delays CCR5 internalisation and blocks stimulation by human chemokine

**Figure 4:**
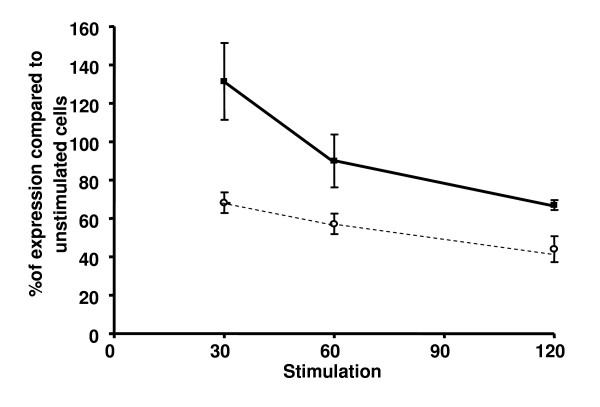
**U83A treatment results in long-term delays of CCL4 driven internalisation of CCR5 in the absence of re-stimulation**. CCR5 expression in MAGI-CCR5 cells was monitored by flow cytometry after incubation with 100 nM U83A in buffer (plain line) or control, buffer only (dashed line) for 10 minutes, then washed, incubated for the indicated time (x axis, in minutes), and stimulated with 100 nM CCL4 for 10 minutes, cells were then stained with FITC-CCR5 antibody and fixed. CCR5 surface levels were then determined by flow cytometry. Results were normalised in reference to CCR5 level of expression at time 0, as 100% values, and are a combination of two experiments each run in duplicate. P values for mean differences are 0.029, 0.066 and 0.016 for 30, 60 and 120 minutes time points, respectively (unpaired T test).

Next investigated, was the persistence of U83A in delaying internalisation of signalling receptors after endogenous human chemokine stimulation, thus re-directing these chemokines towards their clearance by scavenger receptors. We have shown previously that CCR5 expression at the cell surface was not altered by U83A treatment, with minimal effects up to an hour, in contrast to stimulation with human chemokines which showed rapid, clathrin mediated internalisation after 5 minutes treatment [8]. This time, cells expressing CCR5 were incubated in absence or presence of U83A, then washed and incubated for 30, 60 and 120 minutes before being challenged with CCL4 for 10 minutes at each time point. CCR5 surface expression was then determined by flow cytometry. Figure 4 shows for the first time, that even after washing (therefore in the absence of continual re-stimulation), cell bound U83A durably inhibits CCL4 induction of CCR5 internalisation for up to 2 hours after the initial incubation with U83A. Our previous results had demonstrated delayed CCR5 internalisation in the presence of U83A [8]. Here, since the unbound U83A had been washed off before stimulation by CCL4, this new observation demonstrates continued inhibition of CCR5 internalisation in the absence of continuous U83A stimulation. In contrast, the control experiment, in the absence of U83A pre-treatment, showed a normal rapid internalisation of CCR5 surface expression after 10 minutes CCL4 stimulation. Treatment with U83A-N, only showed CCR5 internalisation delay at 30 minutes, the other time points were not significant (Table 2).

**Table 2:** Percentage of CCR5 surface expression after U83A or U83A-N treatment followed by CCL4 stimulation

Pretreatment:	Buffer			U83A			U83A-N		
Time CCL4 stimulation	%CCR5	SEM	N	% CCR5	SEM	N	%CCR5	SEM	N

30 minutes	68.2	5.4	4	131.4	20.1	4	90.6	7.2	4
60	57.1	5.3	4	90.0	13.8	4	74.2	16.0	4
120	44.0	6.8	3	66.95	2.6	4	46.6	5.6	4

SEM: Standard error of mean, N: replicates

### Diversion by U83A of CCR5 internalisation via delayed caveolin linked pathway

**Figure 5:**
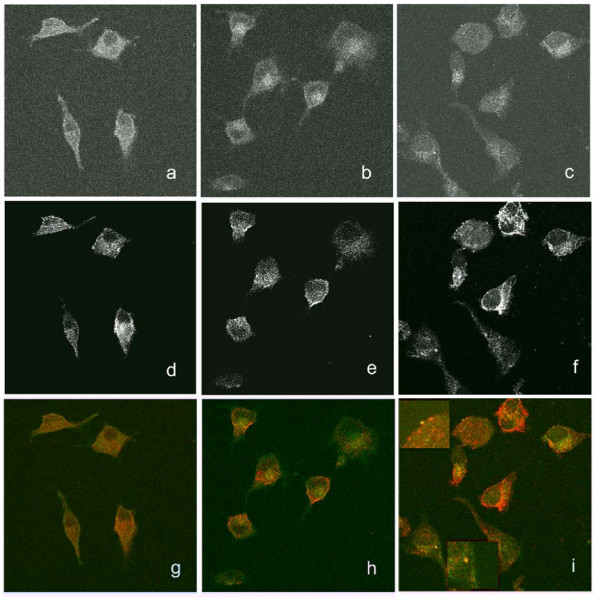
**U83A delays internalisation of CCR5 and effects on localisation of caveolin-1**. Localisation of CCR5 and caveolin-1 were monitored by confocal microscopy in the absence of chemokine stimulation, buffer only (a, d, g), and when stimulated for 10 minutes with CCL4 (b, e, h) or U83A (c, f, i). DAPI nucleus staining was used as a control, which gave nuclear staining of all cells indicated (not shown). After stimulation, cells were fixed and permeabilised, then reacted with CCR5 antibody linked to FITC, green channel (a, b, c) showing internalisation induced by CCL4 (b), and caveolin-1 antibody linked with TRITC, red channel (d, e, f), showing coalescence of punctuate staining induced by U83A (f). The merged staining (g, h, i) shows for U83A treatment, examples of punctae of caveosome-like structure, with yellow merged fluorescence as indicated in the enlarged insets (i). Representative 0.39 micron slices are from the z-stack from three independent assays.

**Figure 6:**
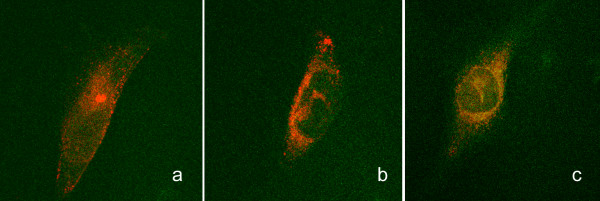
**U83A specific induction of co-localisation of CCR5 with caveolin-1**. Localisation of CCR5 and caveolin-1 was monitored by confocal microscopy, in absence of stimulation (a), when stimulated with 30 minutes with 100 nM CCL4 (b) or U83A (c). CCR5 is labelled with antibody linked to FITC (green) and caveolin-1 with TRITC (red), co-localisation appears as yellow.

Previous results showed that treatment with U83A did not link CCR5 with clathrin [8], as opposed to that described for endogenous chemokine stimulation of CCR5 in similar cellular models [23] which clearly show clathrin linked endocytosis even after 5 minutes treatment. In contrast, as shown here, after 10 minutes treatment with U83A, there was little effect on CCR5 redistribution, although coalesced punctate staining of caveolin-1 was observed (Figure 5f). This pattern was distinct from treatment with the human chemokine CCL4, specific for CCR5, which showed CCR5 internalisation with clustering at the centre of the cells (Figure 5b), but only limited effects on caveolin staining. There was no evidence of co-localisation of CCR5 with caveolin-1 for either CCL4 of buffer treatments. In contrast, cells treated with U83A showed some isolated punctae of CCR5 co-localised with caveolin-1 (Figure 5i). After further 30 min stimulation with U83A, where there was more advanced internalisation, caveolin-1 was clearly linked with CCR5 in a caveosome-like vesicular array (Figure 6c). Taken together, these results show that not only U83A delays the internalisation but it also hijacks CCR5 receptors to caveolin rich membrane domains, implicating diversion toward different internalisation or signalling pathways (Figure 4).

## Discussion

Results show that U83A exercises a complex and thorough control of CCR5 signalling receptor activity, while bypassing clearance by D6 or DARC regulatory receptors (Table 1). This could account for unregulated U83A activity in chronic inflammatory disease linked with HHV6-A, including neuroinflammatory pathology such as MS and encephalitis [24,25].

D6 is involved in a rapid and constant constitutive internalisation and degradation of circulating human chemokines. Binding of chemokine to D6 does not result in the activation of major chemokine signalling pathways and thus restrains the inflammatory process by competition. A role of D6 in MS has been indicated through its involvement in chemokine clearance to regulate inflammation as well as alteration of immune cell localisation hence impairment of immune function [26]. D6 expression has been demonstrated on lymphatic endothelial cells, in skin, gut and lungs with roles as a chemokine sequestering decoy [12]. It is also implicated in clearance of chemokines in placenta; D6^-/- ^mice show increased miscarriage indicating D6 expression protective [13,27]. Furthermore, congenital and placental infections with HHV-6A/B have been demonstrated as well as virus reactivation during pregnancy [28,29]. Case reports show infections in rare seronegative women with spontaneous abortion and neuro-inflammatory complications in the newborn after HHV-6 transplacental infection, and primary infected infants [30-32].

DARC can be considered a chemokine buffer, acting as a chemokine reservoir when expressed on erythrocytes [33]. It is also expressed on vascular endothelial cells and a role in transcytosis, supporting leukocyte migration, has been proposed; it is up-regulated in several inflammatory diseases [17,34-37]. DARC upregulation has been associated with acute renal transplant rejection [35], and co-localisation of DARC and CCR5 expressing cells has been suggested as a common process during graft rejection, with implications for HHV-6 association with acute renal graft rejection [38]. Here we show U83A can avoid DARC but still attract CCR5 expressing cells. Unregulated U83 may drive other inflammatory pathologies, such as HHV-6 associated myocarditis [39,40], since autoimmune myocarditis is escalated by CCR5-bearing activated T-cells and monocytic/macrophages [41], which can be chemoattracted by U83A.

Multifaceted interactions of CCR5 with caveolin rich membrane regions (or rafts) are suggested in a recent report [42] which shows that signalling induced by receptors expressed in these regions differs from signalling induced by receptors expressed elsewhere. Raft domains are often described as favouring the interactions between surface expressed receptors and intracellular activation pathways, (e.g CXCR1 partitioning to lipid raft is assumed to enhance its activity [43]). However it is likely that it only modifies the coupling rather than induces it, as for example CCR5 can signal in absence of raft [42]. At the virus level, it is also noteworthy that U51, one of the HHV6 encoded chemokine receptors which binds and is activated by CCL5, can establish unusual coupling to G proteins. U51 can induce pertussis toxin (PTX)-insensitive increases in phospholipase C activity and changes in intracellular free calcium concentration, which are Gq modulated, while different ligands can re-direct to a Gαi linked pathway [10]. Gathering CCR5 to a caveolin raft where Gq coupling is favoured [44], might also facilitate U51 coupling to Gαi elsewhere. Thus redirecting CCR5 to a raft membrane microdomain, can be another pathway to control its activity to avoid its internalisation and to direct signalling.

Finally, extended CCR5 blocking could lead to enhanced U83A displacement of HIV-1 from the co-receptor CCR5, since only the human chemokines which also compete, such as CCL5, can be cleared by DARC or D6. This would also enhance the competitive inhibition of HIV-1 infection we have previously demonstrated by U83A [8]. To our knowledge this internalisation inhibition by a heterologous chemokine is unique. CCR5 is a key signalling molecule to infection. For HIV-1 it not only serves as co-receptor, but activation via CCR5 is important in development of an efficient immune response to the infection [45,46]. CCR5 is expressed on plasmacytoid and immature myeloid dendritic cells, plus monocytic/macrophages, NK cells as well as key T cell subsets such as TH1, naive CD8, and some Treg cells, thus important in protective inflammatory responses in infected tissue sites [15,47]. It is essential for developing a TH1 cell response, which controls intracellular virus infections. Although deletion of CCR5 surface expression as observed in the CCR5delta32 mutation provides protection against HIV-1 infection, blocking as well as stimulation of CCR5 via efficient human chemokine CCL3L1 can also enhance protection, similar to the activities shown here by viral U83A chemokine [45]. Further, CCR5 expression promotes resistance to West Nile Virus infections [48]. In HHV-6A, CCR5 effects are targeted on multiple levels. U83A displaces human chemokines from binding, and also delays internalisation and hence recycling of unbound CCR5. It diverts CCR5 to signalling via a caveolin-linked pathway, which still allows chemotaxis, but delays signalling via human chemokines, hence only recruitment of cells susceptible for infection rather than activated cells for immunity and clearance. The displacement of human chemokines, both by competitive binding and delayed internalisation preventing restimulation, can act as a co-factor to DARC and D6, which can bind and sequester these human ligands of CCR5. This would raise the levels of chemokines recognised by D6, which can induce its membrane expression and further enhance chemokine degradation [49]. Furthermore, HHV-6A U51 chemokine receptor can both bind and downregulate expression of CCL5, which normally interacts with CCR5. To sum up, U83A permits continued sequestering of human chemokines via regulatory receptors away from an infectious centre, while occupying the human signalling receptors, preventing their physiological recycling, directing CCR5 to a different internalisation pathway and displacing normal interactions with endogenous chemokines.

U83A effects add to the increasing evidence for pivotal roles played by receptor internalisation regulation in the complexity of cell responses to chemokines. In contrast to the full-length U83A effects, the spliced truncated U83A-N, had lower affinity binding, did not mediate specific chemotaxis, and had no significant effects on internalisation. Similarly, CCR7 is differentially internalized according to the stimulating ligand (CCL19 or CCL21), suggesting different functions or regulation of these two otherwise redundant chemokines [50]. While differential internalisation efficiency of CCR3 by different chemokines was also demonstrated with eotaxin/CCL11 versus RANTES/CCL5 [51]. Recent results also show further inhibition mechanisms, where dimerisation of DARC with receptor CCR5 prevents chemotaxis without affecting internalisation [52]. This inhibitory effect would also be amplified with U83A interaction with CCR5 preventing recycling and exposure to signalling chemokines.

## Conclusion

HHV6 U83A has developed a double ability to escape clearance by avoiding regulatory scavenger chemokine receptors and by delaying internalisation/recycling of their classical target signalling receptors, therefore together with previously defined agonist activities and complementary effects of the virus chemokine receptor, these actions provide molecular mechanisms for linked inflammatory pathologies, such as MS, as well as applications for novel immunomodulators.

## Competing interests

The authors declare that they have no competing interests.

## Authors' contributions

JC carried out the binding, internalisation and confocal microscopy studies, and contributed to drafting the manuscript. DJC carried out human leukocyte culture and chemotaxis flow cytometry assays. UAG participated in the design and analyses of the study and drafting the manuscript. All performed statistical analysis of the data. All authors read and approved the final manuscript.
